# Consumption of Diet Containing Free Amino Acids Exacerbates Colitis in Mice

**DOI:** 10.3389/fimmu.2017.01587

**Published:** 2017-11-20

**Authors:** Adna Luciana Souza, Sarah Leão Fiorini Aguiar, Mariana Camila Gonçalves Miranda, Luisa Lemos, Mauro Andrade Freitas Guimaraes, Daniela Silva Reis, Patrícia Aparecida Vieira Barros, Emerson Soares Veloso, Toniana Gonçalves Carvalho, Fabiola Mara Ribeiro, Enio Ferreira, Denise Carmona Cara, Ana Cristina Gomes-Santos, Ana Maria Caetano Faria

**Affiliations:** ^1^Departamento de Bioquímica e Imunologia, Universidade Federal de Minas Gerais, Belo Horizonte, Brazil; ^2^Centro das Ciências Biológicas e da Saúde, Universidade Federal do Oeste da Bahia, Barreiras, Brazil; ^3^Departamento de Patologia Geral, Instituto de Ciências Biológicas, Universidade Federal de Minas Gerais, Belo Horizonte, Brazil; ^4^Departamento de Morfologia, Instituto de Ciências Biológicas, Universidade Federal de Minas Gerais, Belo Horizonte, Brazil; ^5^Centro Universitário UNA, Belo Horizonte, Brazil

**Keywords:** nutrition, amino acids, colitis, gut homeostasis, mammalian target of rapamycin

## Abstract

Dietary proteins can influence the maturation of the immune system, particularly the gut-associated lymphoid tissue, when consumed from weaning to adulthood. Moreover, replacement of dietary proteins by amino acids at weaning has been shown to impair the generation of regulatory T cells in the gut as well as immune activities such as protective response to infection, induction of oral and nasal tolerance as well as allergic responses. Polymeric and elemental diets are used in the clinical practice, but the specific role of intact proteins and free amino acids during the intestinal inflammation are not known. It is plausible that these two dietary nitrogen sources would yield distinct immunological outcomes since proteins are recognized by the immune system as antigens and amino acids do not bind to antigen-recognition receptors but instead to intracellular receptors such as mammalian target of rapamycin (mTOR). In this study, our aim was to evaluate the effects of consumption of an amino acid-containing diet (AA diet) versus a control protein-containing diet in adult mice at steady state and during colitis development. We showed that consumption of a AA diet by adult mature mice lead to various immunological changes including decrease in the production of serum IgG as well as increase in the levels of IL-6, IL-17A, TGF-β, and IL-10 in the small and large intestines. It also led to changes in the intestinal morphology, to increase in intestinal permeability, in the number of total and activated CD4+ T cells in the small intestine as well as in the frequency of proliferating cells in the colon. Moreover, consumption of AA diet during and prior to development of dextran sodium sulfate-induced colitis exacerbated gut inflammation. Administration of rapamycin during AA diet consumption prevented colitis exacerbation suggesting that mTOR activation was involved in the effects triggered by the AA diet. Therefore, our study suggests that different outcomes can result from the use of diets containing either intact proteins or free amino acids such as elemental, semielemental, and polymeric diets during intestinal inflammation. These results may contribute to the design of nutritional therapeutic intervention for inflammatory bowel diseases.

## Introduction

Nutrients are important modulators of the immune system and can act in the differentiation of specific subsets of immune cells and in the maintenance of immunological homeostasis (1). Among the nutrients, intact dietary proteins are one of the most critical elements involved in the development and maturation of the immune system after weaning. Although most dietary macromolecules are degraded by the time they reach the small intestine, both in humans and in rodents, some undergraded or partially degraded proteins are absorbed into the blood in an immunogenic form (2–5). Many reports exist showing that small quantities of intact proteins cross the gastrointestinal tract in animals and adult humans at steady state conditions (6).

The specific effect of dietary proteins on the development of immune system has been addressed using either protein-free diets (7) or elemental diets (8) to feed mice after weaning. In both cases, intact dietary proteins were replaced by equivalent amounts of free amino acids as nitrogen source and they were shown to influence the maturation of the immune system, particularly the gut-associated lymphoid tissue when consumed from weaning to adulthood (7). Moreover, the presence of intact dietary proteins after weaning is important for the generation of regulatory T cells in the small intestine (8) and for the full development of immune activities during adulthood such as a protective response to infection (9), induction of nasal (10) and oral tolerance as well as allergic reactions (11). Thus, the role of intact dietary proteins in the maturation of immunological activity at the early stages of life is clear. However, the influence of this nutrient in adult animals when the immune system is already mature is not known.

To understand the immune effects of either intact proteins or free amino acids as nitrogen sources is of great importance in clinical practice, especially in cases of intestinal inflammation. It has been reported that nutritional intervention plays a beneficial role in the treatment of inflammatory bowel diseases (IBDs). Polymeric diets, which contain intact proteins, usually yield better results than elemental formulas with only free amino acid (12–14), but the specific effects of different nitrogen sources in these inflammatory conditions is still elusive.

Several types of diet interventions have been demonstrated to ameliorate inflammation in IBD patients. Suskind et al. showed that a specific carbohydrate diet (SCD) therapy during the course of Crohn’s is associated with improvement in clinical and laboratory parameters as well as with concomitant changes in the fecal microbiome (15, 16). Lewis et al. also showed that exclusive enteral nutrition (EEN) with an elemental diet has a positive impact on both microbiota composition and Crohn’s disease development (17). In addition, Zhan et al., in a meta-analysis, concluded that diets containing low fermentable oligosaccharides, disaccharides, monosaccharides, and polyol are beneficial for reducing gastrointestinal symptoms in patients with quiescent IBD (18).

Thus, the aim of this study is to clarify the effects of consumption of diets containing free amino acids during adulthood in gut homeostasis at steady state and under inflammatory conditions. Our results may contribute to understand the direct and side effects of elemental, semielemental, and polymeric diets used for the treatment of intestinal conditions such IBDs.

## Materials and Methods

### Animals

Male C57BL/6 mice used in the experiments were obtained from the Animal Breeding Facility (CEBIO) of Universidade Federal de Minas Gerais (UFMG). Mice were maintained under environmentally controlled conditions (using Alesco^®^ racks with individually ventilated cages) with a 12-h light–dark cycle at the experimental animal facility of Laboratório de Imunobiologia, Instituto de Ciências Biológicas, UFMG, Belo Horizonte, Brazil. Different experimental groups were housed in separate cages. At the end of the experiments, mice were sacrificed by cervical dislocation. All procedures were approved by the Ethics Committee for Animal Use in Research of UFMG (Protocol no. 114/2010, CEUA-UFMG, Brazil).

### Diets

Experimental diets were prepared using the AIN 93M standard formulation (19) with modifications in the nitrogen source. Experimental diet containing free amino acids (AA diet) and control diet contained whole casein (CAS diet) at equivalent concentrations. Diets were isocaloric and identical with respect to all other nutrients (Table 1). Amino acids used for the experimental AA diet were the same found in casein (Table 2) at similar ratios.

**Table 1:** Composition of the control diet (CAS diet) and the experimental diet containing free amino acids (AA diet).

Components (g/100 g)	Control diet (casein)	Experimental diet (AA)
Casein	17.9	–
Free amino acid	–	17.9
Corn starch	39.75	39.75
Dextrinized corn starch	13.2	13.2
Sucrose	10.0	10.0
Soybean oil	7.0	7.0
Cellulose	5.0	5.0
Mineral mix	3.5	3.5
Vitamin mix	1.0	1.0
Supplementation of l-cystine	0.3	0.3
Choline bitartrate	0.25	0.25
Tert-butylhydroquinone	0.0014	0.0014

*Diet composition is presented by g/100 g and it is in accordance with AIN 93G diets*.

*Total energy 376.7 kcal/100 g; 16.7% fat; 64% carbohydrates; and 19.3% nitrogen source (casein or amino acid)*.

**Table 2:** Amino acid composition of AA diet (modified AIN 93G).

Amino acid composition (g/100 g)			
Alanine	0.46	Lysine	1.3
Arginine	0.64	Methionine	0.46
Aspartic acid	1.22	Phenylalanine	0.88
Cysteine	0.37	Proline	2.05
Glutamic acid	3.63	Hydroxiproline	<0.1
Glycine	0.32	Serine	0.97
Histidine	0.46	Threonine	0.67
Isoleucine	0.85	Tryptophan	0.21
Leucine	1.54	Tyrosine	0.93
		Valine	1.0

### Diet Administration at Steady State Conditions

**Figure 1:**
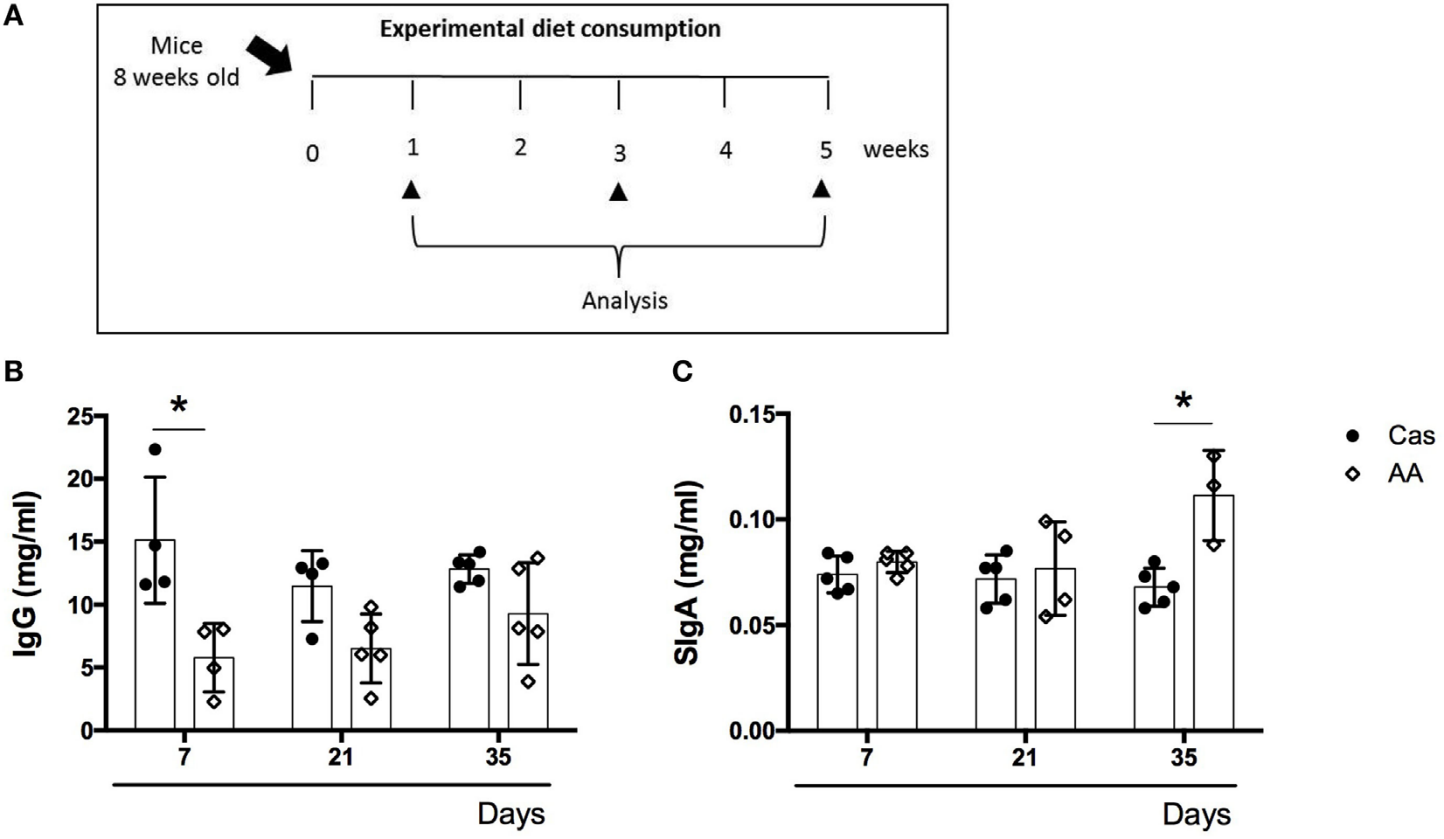
Effects of a diet containing free amino acids (AA diet) on immunoglobulin production. **(A)** C57BL/6 mice at 7–8 weeks of age were fed either experimental diet (AA diet) or control diet [casein-containing diet (CAS) diet)]. Administration of diets was maintained for 5 weeks. Levels of total serum immunoglobulins **(B)** and SIgA in the colon **(C)** were measured at the end of 7, 21, and 35 days of diet consumption by enzyme-linked immunosorbent assay (ELISA) (*n* = 5). Statistical analysis was performed between CAS-fed and AA-fed group at the end of each experimental time point using Student’s *t* test for parametric data. *p* < 0.05.

C57BL/6 mice at 7–8 weeks of age were fed either experimental diet or control diet for 5 weeks (Figure 1A). Water was offered *ad libidum*.

### Diet Administration during Colitis

In the first protocol, C57BL/6 mice at 7–8 weeks were introduced to either experimental diet or control diet during colitis development. Colitis was induced by three cycles of 1% (w/v) dextran sodium sulfate (DSS; 40 kDa) administration in drinking water for 7 days, alternating with 7-day periods of recovery. Diet consumption and the first DSS administration started at the same time. Control group received only water. Liquid consumption was monitored and all mice groups consumed similar volumes of DSS solution daily. After the last DSS cycle, animals were euthanized.

The second protocol was designed to evaluate the lasting effects of these diets when consumed previously to colitis induction. Mice received either amino acid or casein diet for 7 days. Experimental diets were then replaced by commercial chow and colitis was induced by 1% DSS consumption for 7 days.

### Rapamycin Treatment

To evaluate the role of mammalian target of rapamycin (mTOR) activation by AA diet in colitis, mice received daily 200 μl rapamycin (at 10 mg/ml per mice) intraperitoneally for 7 days during diet consumption and prior to colitis induction.

### Biochemical Tests

Total serum proteins, albumin, transferrin (Labtest^®^, Lagoa Santa, MG, Brazil), glycemia (Katal^®^, Belo Horizonte, MG, Brazil), serum triglycerides, and cholesterol (Doles^®^, Goiânia, GO, Brazil) were determined using enzymatic kits.

### Intestinal Lavage Samples

For measurement of secretory IgA (sIgA), intestinal contents of the small and large intestines were collected by carefully washing the intestinal lumen of each portion of the intestine with 10 and 5 ml of cold PBS, respectively. Collected material was transferred to a test tube, vigorously vortexed, and centrifuged for 30 min at 850 *g* at 4°C. The supernatant was transferred to a new test tube and freshly tested by enzyme-linked immunosorbent assay (ELISA) for IgA concentration as previously described (20).

### Measurement of Secretory IgA and Total Serum Immunoglobulins

Levels of isotype-specific Ig in intestinal lavage samples and serum were determined by ELISA. Briefly, 96-well plates (Nunc^®^, Sigma-Aldrich, St. Louis, MO, USA) were coated with 0.1 mg/ml goat antimouse Ig in coating buffer, pH 9.8. Wells were blocked with 200 µl PBS contain 0.25% casein for 1 h at room temperature. After washing the plates six times, serial dilutions of samples were added to wells and incubated for 1 h at 37°C. Plates were washed six times again and 100 µl biotinylated goat antimouse heavy chain-specific polyclonal antibodies were added, and then incubated for 1 h at 37°C. After six washes, a detection solution containing a 1/10,000 dilution of horseradish peroxidase-conjugated streptavidin was added and incubated for 45 min. After washing, color reaction was developed at room temperature using 100 μl/well orthophenylenediamine (1 mg/ml), 0.04% H_2_O_2_ substrate in sodium citrate buffer. Reaction was interrupted by the addition of 20 μl/well 2 N H_2_SO_4_. Absorbance was measured at 492 nm by an ELISA reader (BIO-RAD, Philadelphia, PA, USA) and Ig concentrations were determined using a standard curve.

### Intestinal Tissue Preparation and Cytokine Assay

Colon and small intestine samples were weighed and homogenized in PBS containing 0.05% Tween-20, 0.1 mM phenylmethylsulphonyl fluoride, 0.1 mM benzethonium chloride, 10 mM ethylenediaminetetraacetic acid (EDTA), and 20 KIU Aprotinin A using a tissue homogenizer (100 mg tissue/ml buffer). Suspensions were centrifuged at 12.000 *g* for 20 min at 4°C and the supernatants were transferred to microtubes and stored at −80°C until analysis. Supernatants were collected to assess cytokine levels of IL-6, IL-12, IL-4, IL-10, IL-17A, and TGF-β by capture ELISA. Briefly, plates were coated with purified monoclonal antibodies reactive with IL-6, IL-12, IL-4, IL-10, IL-17A, and TGF-β (BD-Pharmingen, Franklin, NJ, USA) overnight at 4°C. For TGF-β analysis, only active TGF-β was measured; no acidification step was performed. In the following day, wells were washed, supernatants were added, and plates were incubated overnight at 4°C. In the third day, biotinylated monoclonal antibodies against cytokines were added and plates were incubated for 2 h at room temperature. Color reaction was developed at room temperature with 100 μL/well orthophenylenediamine (1 mg/mL), 0.04% H_2_O_2_ substrate in sodium citrate buffer. Reaction was interrupted by the addition of 20 μl/well 2 N H_2_SO_4_. Absorbance was measured at 492 nm by ELISA reader (BIO-RAD, Philadelphia, PA, USA).

### Cell Preparation and Flow Cytometry Analysis

Following euthanasia, small intestine and colon tissues were harvested; mesenteric and adipose tissues were removed. Visible Peyer’s patches were removed. Tissues were then cut open longitudinally and drawn through a pair of curved forceps while applying gentle pressure to remove intestinal contents. Tissues were cut into 2–4 cm fragments, then washed twice to remove feces in calcium- and magnesium-free HBSS containing 2% FCS (at 4°C). Tissues were placed in 50-ml tubes and washed three times in HBSS containing 2% FCS at 4°C. Tissues were transferred to 25-cm^3^ tissue culture flasks and incubated at 37°C in HBSS containing 10% FCS, 0.2 mmol/l EDTA, 1 mmol/l DTT, 100 U/ml penicillin, and 100 µg/ml streptomycin. After 20 min, flasks were shaken vigorously for 30 s, and the supernatant containing the intraepithelial lymphocytes was separated from the tissue fragments using a stainless steel sieve. To isolate *lamina propria* lymphocytes, the remaining tissue was washed three times with cRPMI, and intestinal pieces were subsequently incubated for 30 min at 37°C in cRPMI supplemented with 100 U/mL liberase. Cells were separated from tissue debris by purification through a 70-µm nylon filter. This step was repeated with a 40-µm nylon filter. Cell suspensions were adjusted to 106 cells/ml. For surface antigen detection, cells were labeled with monoclonal antibodies (anti-CD4 FITC, anti-CD44 PE, anti-IL-23 Percep 5.5) for 30 min at 4°C. For intracellular labeling, a fixing kit/permeabilization (e-Bioscience, San Diego, CA, USA) was used after this step. Then samples were incubated for 30 min with either PE-labeled anti-Foxp3 or anti-RORγt antibodies. After washes with PBS-wash, samples were fixed with 3% paraformaldehyde for 30 min, washed and stored in PBS at 4°C. Cells were acquired using a FACSCanto II cytometer (Becton & Dickinson, East Rutherford, NJ, USA) and data were analyzed by FlowJo software (TREESTAR, Ashland, OR, USA).

### Histomorphometrical Analysis

Histological sections of small intestine and colon were obtained from casein-fed and AA-fed mice. Tissues were fixed by 10% PBS-buffered formalin, embedded in paraffin, and 3-mm thick sections were obtained, stained with hematoxylin and eosin and examined under a light microscope. To measure the thickness of crypts, mucous and muscular layer, villus height and goblet cell numbers, the selected image was focused by an optical microscope and captured by a video camera JVC TK-1270/RGB previously coupled to the microscope body. The captured image was digitalized, transferred to a microcomputer and analyzed using the software *Image J*. For each parameter, at least 100 cells by field were counted.

### Histological Score

Histological examination was performed using a blind score based on a semiquantitative system described previously (21). Each score was determined as follows: extent of destruction of normal mucosal architecture (0: normal; 1: mild; 2: moderate; 3: extensive damage), presence and degree of cellular infiltration (0: normal; 1: mild; 2: moderate; 3: transmural infiltration), extent of muscle thickening (0: normal; 1: mild; 2: moderate; 3: extensive thickening), presence or absence of crypt abscesses (0: absent; 1: present), and the presence or absence of goblet cell depletion (0: absent; 1: present). Scores for each feature were summed up to a maximum possible score of 11.

### Clinical Score

Clinical evaluation was assessed using a previously defined scoring system, which includes loss of body weight, diarrhea, and the presence of blood in the stools. Each score was determined as follows: change in weight (0: <1%; 1: 1–5%; 2: 5–10%; 3: 11–15%; 4: >15%), diarrhea (0: negative; 2: moderate; 4: severe), and stool blood (0: absence; 2: hidden blood; 3: visible blood) as previously described (22). Weight change was expressed as percentage of change in weight compared with the starting weight.

### Western Blotting Analysis

Total and phosphorylated mTOR were evaluated by western blotting analysis. Samples of colon were weighed and homogenized in buffer containing 20 mmol/L Tris-HCl, pH 7.4, 120 mmol/L NaCl, 1 mmol/L EDTA, 5 mmol/L ethylene glycol bis (2-aminoethylether) tetra-acetic acid, 50 mmol/L β-glycerophosphate, 50 mmol/L NaF, 0.3% 3-(3-cholamidepropyl) dimethylammonio-1-propanesulphonate, 1 mmol/L dithiothreitol, 4 mg/mL leupeptin, and 4 mg/mL aprotinin using a tissue homogenizer. The remaining homogenate was centrifuged at 12,000 *g* for 20 min at 4°C. Cell lysates containing equal amounts of protein were separated on 4–12% tris-glycine polyacrylamide gels and then electrophoretically transferred to nitrocellulose membrane. Membranes were incubated with anti-mTOR (L27D4), antiphospho-mTOR (Ser2448), and phospho-p70 S6 kinase (Thr389) specific antibodies (Cell signaling Technology). Then immunoreactive bands were detected using a Western Breeze Chromogenic Western Blot Immunodetection Kit (Invitrogen, Carlsbad, CA, USA).

### Permeability Test

Intestinal permeability was determined by measuring radioactivity diffusion in the blood after oral administration of diethylenetriamine penta-acetic acid (DTPA) labeled with 99 m-technetium (99mTc) as previously described (23). After 7 days of diet consumption, animals received 0.1 mL of a DTPA solution labeled with 18.5 mebequerel (MBq) of 99mTc by gavage. Four hours later, mice were anesthetized, their blood was collected, weighed, and placed in appropriate tubes for radioactivity determination. Blood radioactivity levels were determined using an automated gamma counter (Perkin Elmer Wallac Wizard 1470-020 Gamma Counter; PerkinElmer Inc., Waltham, MA, USA). Obtained results were compared with the standard dose and calculated as a percentage of the dose per gram of blood using the following equation:

$$\text{\%}\;\text{dose}/\text{g blood}=\left(\begin{array}{l} \text{cpm in g of blood}/\\ \text{cpm dose of standard} \end{array}\right)\times 100,$$

where cpm represents the counts of radioactivity per minute.

### Analysis of Proliferating Cells

Analysis of proliferating cells in the intestinal mucosal was performed according to a previously reported methodology (24). Sections of 4 µm were cut from one representative block for each intestinal tissue-containing case and collected on gelatin-coated slides. Slides were deparaffinized and rehydrated in an alcohol series. Endogenous peroxidase was blocked by immersion in 10% hydrogen peroxide. Deparaffinized tissue sections were subjected to heat-induced antigen retrieval (water bath at 98°C) with antigen retrieval solution (DAKO, SA, Denmark, pH 6.0). Next, the slides were incubated at 4°C overnight with a rat monoclonal IgG antiproliferating cell nuclear antigen (PCNA, Clone: PC10, 1:200, Novocastra, Leica Microsystems), followed by the biotinylated link and streptavidin-HRP (LSAB2 System-HRP, Dako, North America) for 10 min each at room temperature. Sections were stained with the chromogen 3,3-diaminobenzidine tetrahydrochloride (DAB Substrate System, Lab Vision), incubated for 1 min and counterstained with Mayer’s hematoxylin.

### Statistical Analysis

Results were expressed as the mean ± standard error of the mean (SEM). All data were analyzed using Kolmogorov–Smirnov normality test to test its Gaussian distribution. Parametric tests (Student’s *t* test and ANOVA) were applied to data with a normal distribution and non-parametric tests (Man–Whitney and Kruskal–Wallis) were used for data that did not follow a normal distribution. To evaluate differences over time, a two-way ANOVA test was used. Means were considered statistically different when *p* < 0.05.

## Results

### Consumption of AA Diet did not Change Nutritional Parameters

Malnutrition affects several aspects of the activity of the immune system (25) and it was important to ensure that the immune alterations observed were not due to nutritional deficiencies. As shown in Figure S1 in Supplementary Material, the two diets (containing either casein or amino acids) were comparable in their ability to maintain the body weight of 8-week-old mice during the 35 days of the experimental analysis (Figure S1A in Supplementary Material). Diet consumption was similar between amino acid- and casein-fed groups (Figure S1B in Supplementary Material). Levels of total serum protein, albumin, glucose, triglycerides, and transferrin were also similar between the two groups (Figures S1C–G in Supplementary Material). These results demonstrate that AA diet was nutritionally equivalent to casein-containing diet (CAS diet) and did not alter the nutritional status of the animals.

### Consumption of AA Diet Led to Reduced Serum IgG Levels and Augmented SIgA Levels in the Colon

Consumption of AA diet did not change the levels of serum immunoglobulins, IgM or IgA (data not shown), but levels of serum IgG were decreased in AA-fed mice after 7 days of diet administration (Figure 1B). There was also a significant increase in the levels of secretory IgA (SIgA) in the colon, but not in the small intestine, of AA-fed mice after 35 days of diet administration (Figure 1C).

### Consumption of Diet Containing Free Amino Acids Affected Cytokine Production

**Figure 2:**
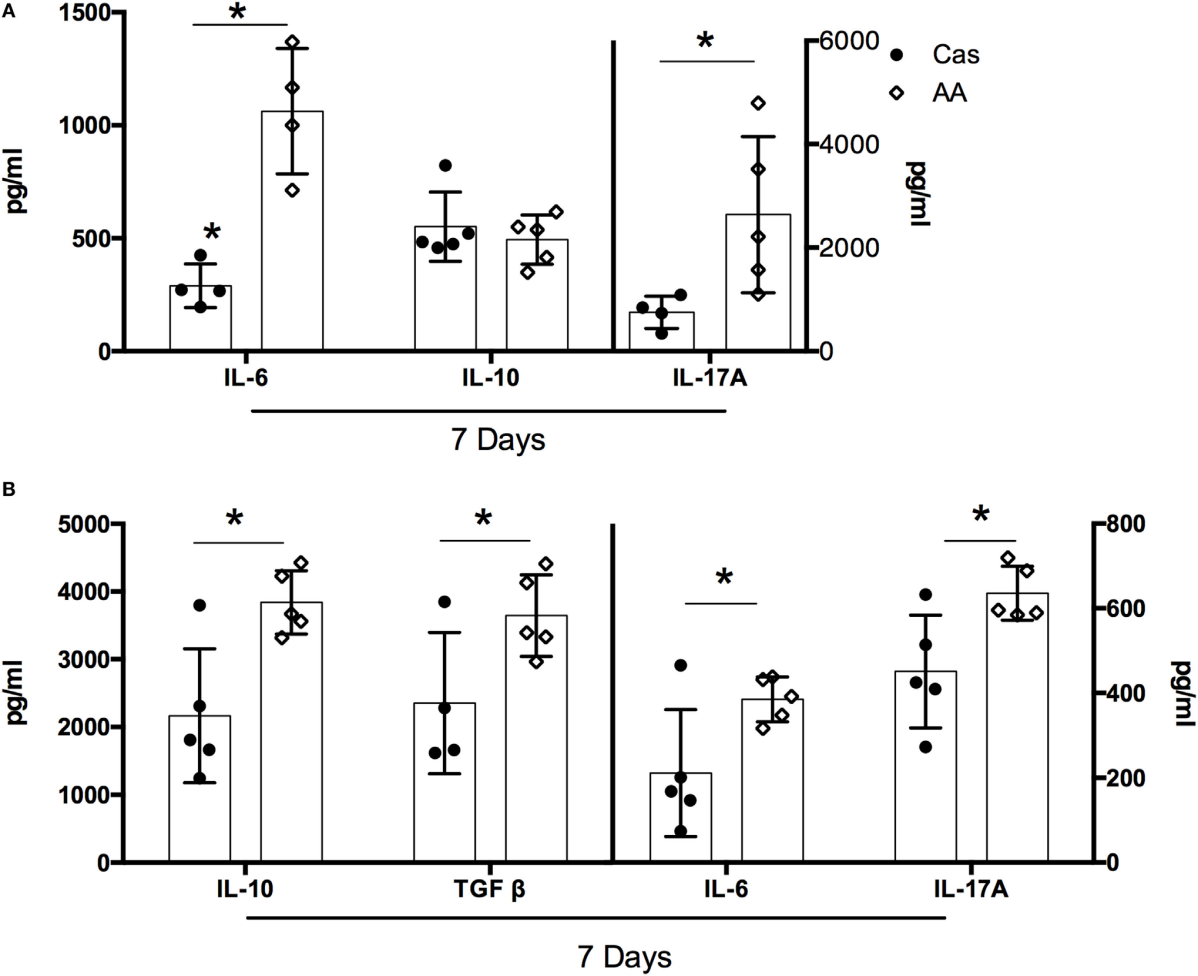
Cytokines levels in the small intestine and colon after dietary consumption. C57BL/6 mice at 7–8 weeks of age were fed either experimental diet [diet containing free amino acids (AA diet)] or control diet [casein-containing diet (CAS diet)] for 5 weeks. Levels of IL-6, IL-17A and IL-10 were measured in the small intestine **(A)**, and levels of IL-10, TGF-β, IL-6, IL-17A, IL-12, IL-4, and IFN-γ were measured in the colon **(B)** by enzyme-linked immunosorbent assay (ELISA) after 7 days of dietary consumption (*n* = 4–7). Statistical analysis was performed between CAS-fed and AA-fed group. *p* < 0.05.

For most of the evaluated cytokines, the effects of AA diet were observed mainly after the first week of consumption. At this time point, AA-fed group showed lower concentrations of IL-10, IL-4, IFN-γ, IL-17A, IL-6, and TGF-β in the spleen, when compared to the casein group (Figure S2 in Supplementary Material). A concomitant increase in cytokine production was found in the intestinal mucosa of AA-fed group. When compared to control CAS-fed mice, AA-fed mice had higher levels of IL-6 and IL-17A in the small intestine (Figure 2A) and higher levels of IL-10, TGF-β, IL-6, and IL-17A in the colon at day 7 of diet consumption (Figure 2B). No alteration in cytokine production was observed between the two groups after either 21 or 35 days of diet administration (data not shown).

### Prolonged Consumption of Diet Containing Free Amino Acids Promoted Increase in the Number of Proliferating Cells and CD4+ T Lymphocytes in the Intestinal Mucosa

**Figure 3:**
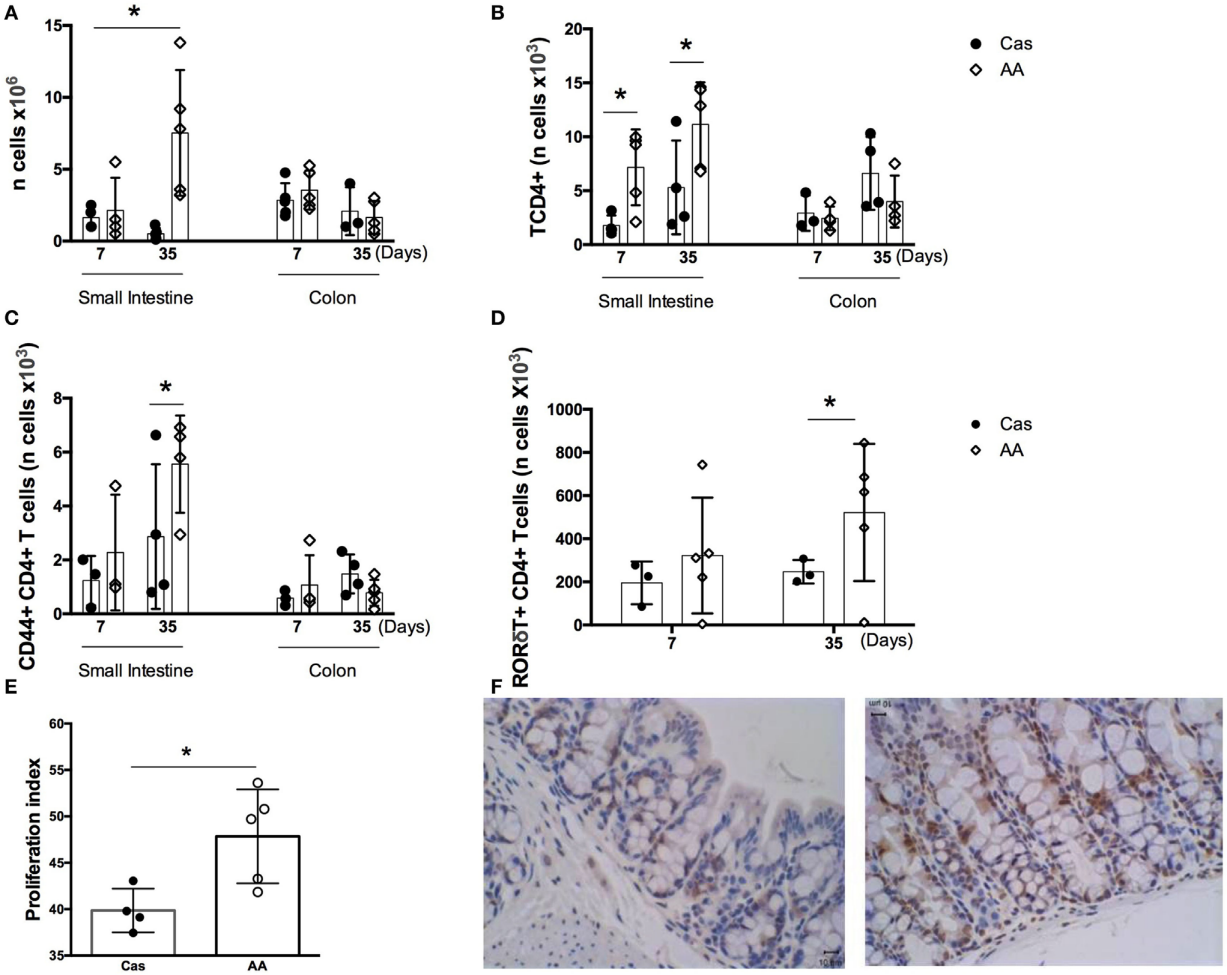
Number of cells in the *lamina propria* of small intestine and colon. C57BL/6 mice at 7–8 weeks of age were fed either experimental diet [diet containing free amino acids (AA diet)] or control diet [casein-containing diet (CAS diet)] for 7 and 35 days. Numbers of total cells **(A)** were determined by cell counting. Numbers of CD4+ T cells **(B)**, CD4 +CD44+ activated T cells **(C)**, and CD4+ RORγt+ T cells in the small intestine **(D)** were determined in the lamina propria by flow cytometry analysis (*n* = 4). Cell proliferation in the small intestine **(E,F)** was evaluated by immunohistochemistry after 7 days of diet consumption. Statistical analysis was performed between CAS-fed and AA-fed group at the end of each experimental time point using Mann Whitney test for non-parametric data. *p* < 0.05.

Dietary replacement of casein by free amino acids led to alterations in the number of cells in the *lamina propria* of gut mucosa. In the small intestine, AA-fed group showed, when compared to the CAS-fed group, an increase in the number of total *lamina propria* cells, CD4+ T lymphocytes, CD44+ CD4+ T lymphocytes, and RORγt+ CD4+ T lymphocytes after 5 weeks of diet consumption (Figures 3A–D). Since the number of lamina propria cells was augmented in AA-fed mice and amino acids are known to signal through intracellular receptors such as mTOR that control cell proliferation, we examined whether proliferative cells were also increased in the small intestinal mucosa using an anti-PCNA monoclonal antibody. AA-fed mice had an augmented number of PCNA+ cells in the small intestine than control CAS-fed mice (Figures 3E–F) suggesting that ingestion of free amino acids had an effect in cell proliferation in the gut mucosa. PCNA is a homotrimer with an essential role in cellular replication by encircling the DNA, where it acts as a scaffold to recruit proteins involved in DNA replication, DNA repair, chromatin remodeling and epigenetics (26). Thus, it is used as reliable marker of proliferating cells. No alteration was found in the number of cells isolated from colons of AA-fed mice.

### Consumption of Free Amino Acids in the Diet Led to Increased Intestinal Permeability and Altered Intestinal Morphology

**Figure 4:**
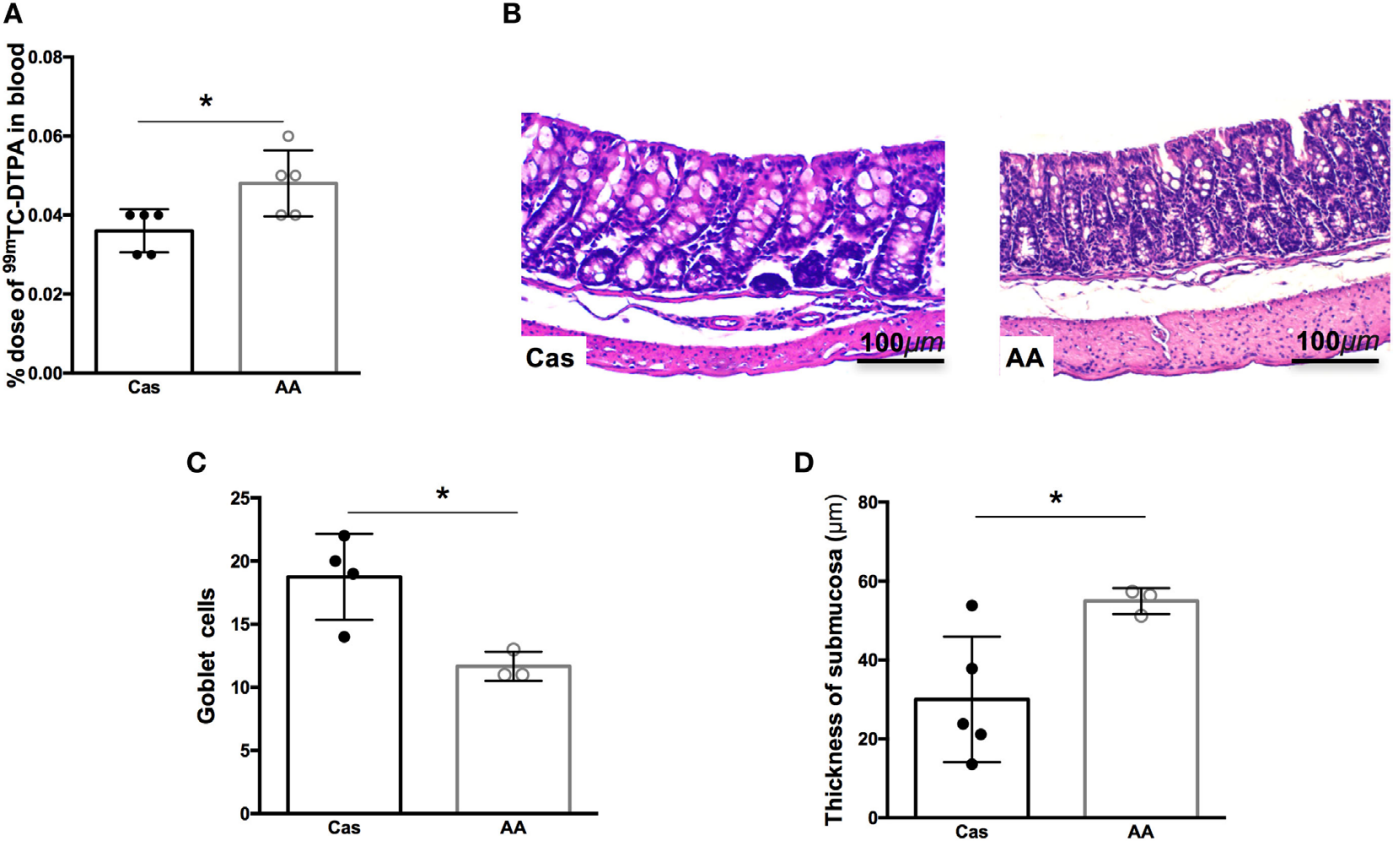
Histological analysis of the small intestine after dietary consumption. C57BL/6 mice at 7–8 weeks of age were fed either experimental diet [diet containing free amino acids (AA diet)] or control diet [casein-containing diet (CAS diet)] for 5 weeks. Intestinal permeability was determined by% dose/g of 99 m-technetium (99mTc)-diethylenetriamine penta-acetic acid (DTPA) in blood **(A)**. Representative images of CAS-fed and AA-fed mice after 35 days of dietary consumption **(B)**. Analysis and measurement of goblet cell numbers **(C)**, and thickness of the submucosa **(D)** were performed in at least three mice per group at 40× magnification. Analysis was performed at 10× magnification. Statistical analysis was performed between CAS-fed and AA-fed group using Mann–Whitney test for non-parametric data. *p* < 0.05.

Since consumption of diet containing free amino acids for 7 days led to changes in cytokine production in the gut, we next investigated whether AA diet could also influence the intestinal permeability and morphology. Intestinal permeability was significantly increased in AA-fed group after 7 days of diet consumption, when compared to casein group (Figure 4A). Intestinal morphology of the colon, but not the small intestine, also showed important alterations. Histopathological changes in the colonic mucosa including increase in cellular infiltration in the mucosal layer, reduction of goblet cell numbers, and increase in the thickness of the submucosal layer were also observed after 5 weeks of AA diet consumption (Figures 4B–D).

### AA Diet Exacerbated DSS-Induced Colitis

**Figure 5:**
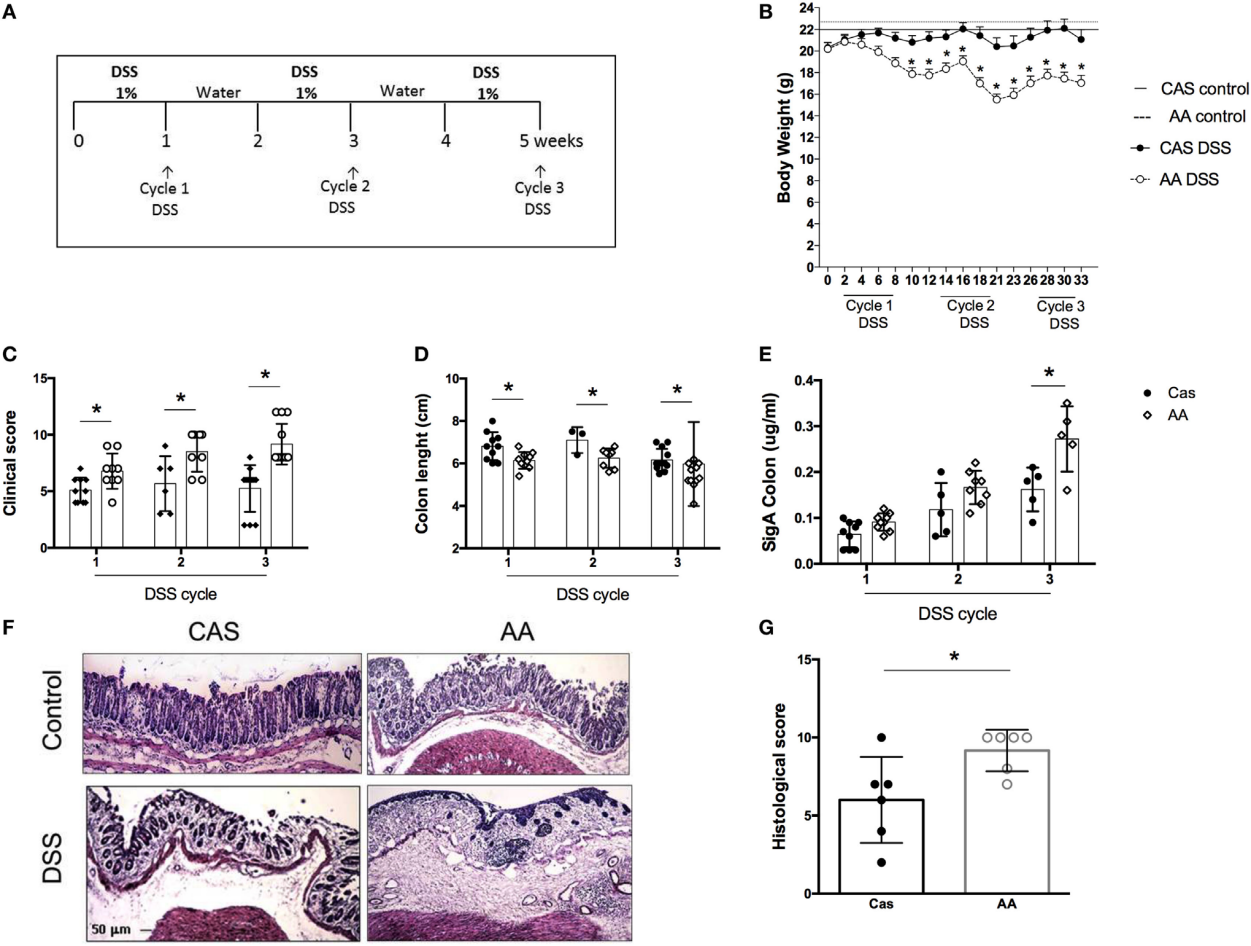
Effects of dietary consumption during dextran sodium sulfate (DSS)-induced colitis development. C57BL/6 mice at 7–8 weeks of age were introduced at experimental diet [diet containing free amino acids (AA) diet] or control [casein-containing diet (CAS)] diet. All mice were kept on these diets throughout colitis induction. Colitis was induced by three-cycle administration of 1% (w/v) DSS in drinking water for 7 days, alternated with 7-day recovery periods **(A)**. Weight loss was evaluated during colitis development **(B)**. Clinical scores of mice with colitis were performed after each cycle of DSS using a scoring system which includes loss of body weight, diarrhea, and presence of blood in the stools **(C)**. Colon length was evaluated after each cycle of DSS **(D)**. SIgA levels were measured in the colonic lavage fluid by enzyme-linked immunosorbent assay (ELISA) **(E)** at the end of each cycle of DSS. Representative images colon of CAS-fed and AA-fed mice **(F)** are shown. Histological scoring **(G)** was performed on samples of the distal colon considering the extent of mucosal architecture destruction, presence and degree of cellular infiltration, extent of muscle thickening, presence or absence of crypt abscesses and presence or absence of goblet cell depletion at the end of the third cycle of DSS (*n* = 5–12). Statistical analysis was performed between CAS-fed and AA-fed group at the end of each experimental time point using Student’s *t* test for parametric data and Mann–Whitney for non-parametric data. *p* < 0.05.

Our results showed that consumption of a diet containing free amino acids was associated with changes in the intestinal mucosa suggesting a local disruption of homeostasis. Our next step was to test whether AA diet would affect intestinal inflammation using a model of colitis induced by the administration of three cycles of DSS (Figure 5A).

AA diet promoted an increase in the severity of colitis. AA-fed mice presented greater weight loss (Figure 5B), more intense bleeding and diarrhea than the CAS-fed mice as evidenced by clinical score at all time points of colitis development (Figure 5C). The length of the colon, which is inversely proportional to the inflammatory status of the tissue, was shorter in the AA-fed mice (Figure 5D). AA-fed mice had also increased levels of SIgA in the colon (Figure 5E) although no change was found in the small intestine. Histological analysis, performed at the end of the third cycle of DSS administration, showed a higher inflammatory score for mice fed the AA diet. This group of mice had greater depletion of goblet cells, erosion of the mucosal layer, more intense cellular infiltrate and edema in the mucosa and submucosal layer (Figures 5F–G). These results demonstrate that consumption of free amino acids during colitis development increased the severity of bowel inflammation.

### Consumption of a Diet Containing Free Amino Acids Prior to Colitis Induction Also Increased the Severity of Gut Inflammation

**Figure 6:**
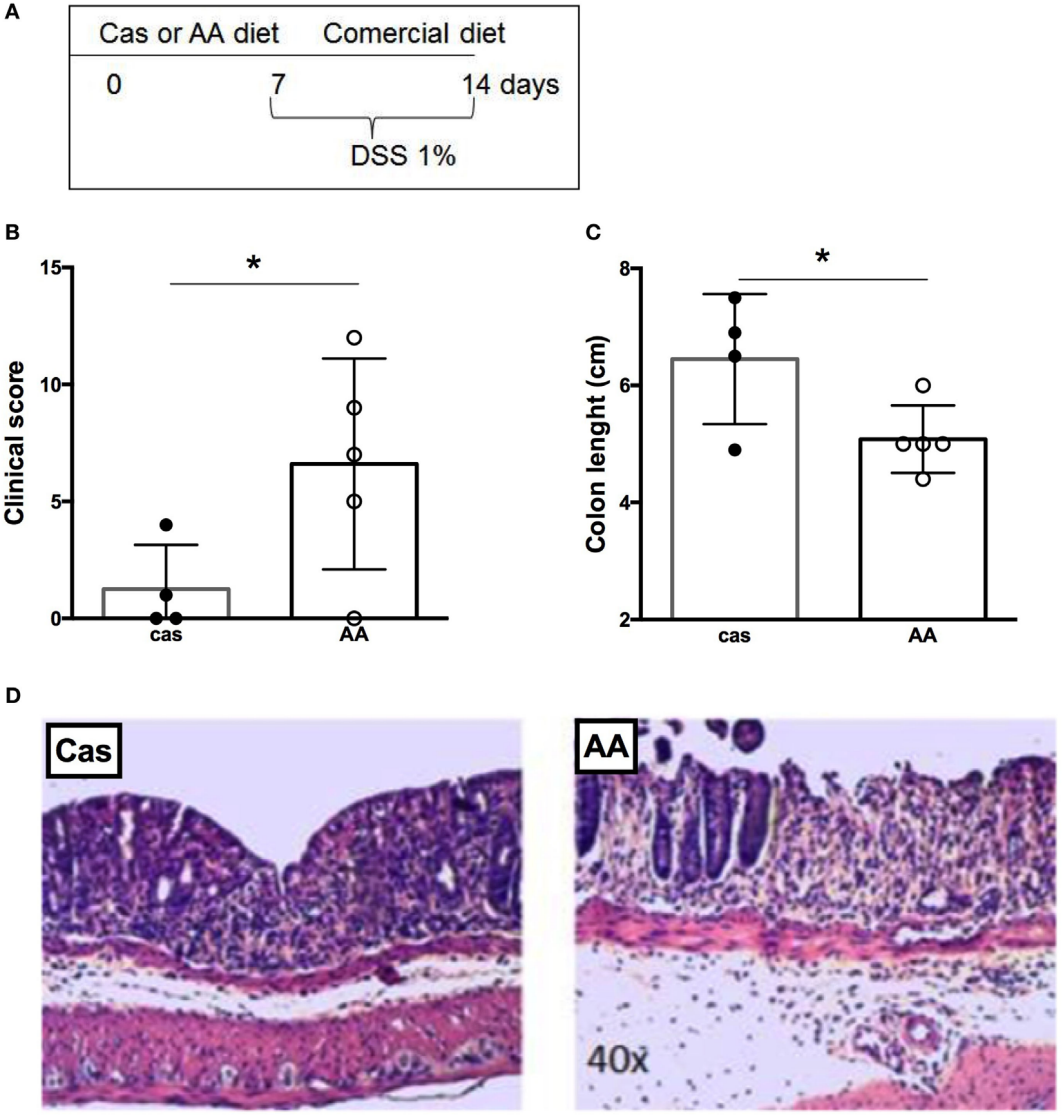
Histological evaluation of colitis development after dietary consumption. **(A)** C57BL/6 mice at 7–8 weeks of age received either casein-containing diet (CAS diet) or diet containing free amino acids (AA diet) for 7 days. Then, experimental diets were replaced by commercial mouse chow and colitis was induced by administration of 1% dextran sodium sulfate (DSS) for 7 days. During colitis development, mice received commercial chow. Clinical scores **(B)** were evaluated after colitis induction using a scoring system, which included loss of body weight, diarrhea, and presence of blood in the stools. Colon length **(C)** was evaluated after the DSS consumption. Representative images of CAS- and AA-fed mice after colitis induction **(D)** are shown. Statistical analysis was performed between CAS-fed and AA-fed group at the end of each experimental time point using Mann–Whitney test for non-parametric data. *p* < 0.05.

We examined next whether AA diet administration prior to colitis induction would also affect disease severity (Figure 6A). Consumption of diet containing free amino acids prior to colitis induction exacerbated gut inflammation. AA-fed mice had a higher clinical score and shorter length of the colon when compared to CAS-fed mice (Figures 6B–D). Thus, the effects induced by diet containing free amino acids in colitis development remained for at least 7 days after diet withdrawal.

### Rapamycin Inhibited the Effects of AA Diet in Colitis Development

Amino acid signaling through the highly conserved serine-threonine kinase mTOR has well-known consequences on proliferation of immune cells and inflammation (27). Therefore, we hypothesized that free dietary amino acids led to increase in inflammation in the gut mucosa by signaling through mTOR in immune cells of the intestinal mucosa. This hypothesis was in concert with the augmented proliferation index found in the intestinal tissues of AA-fed mice when compared to the control CAS-fed group (Figure 3E).

**Figure 7:**
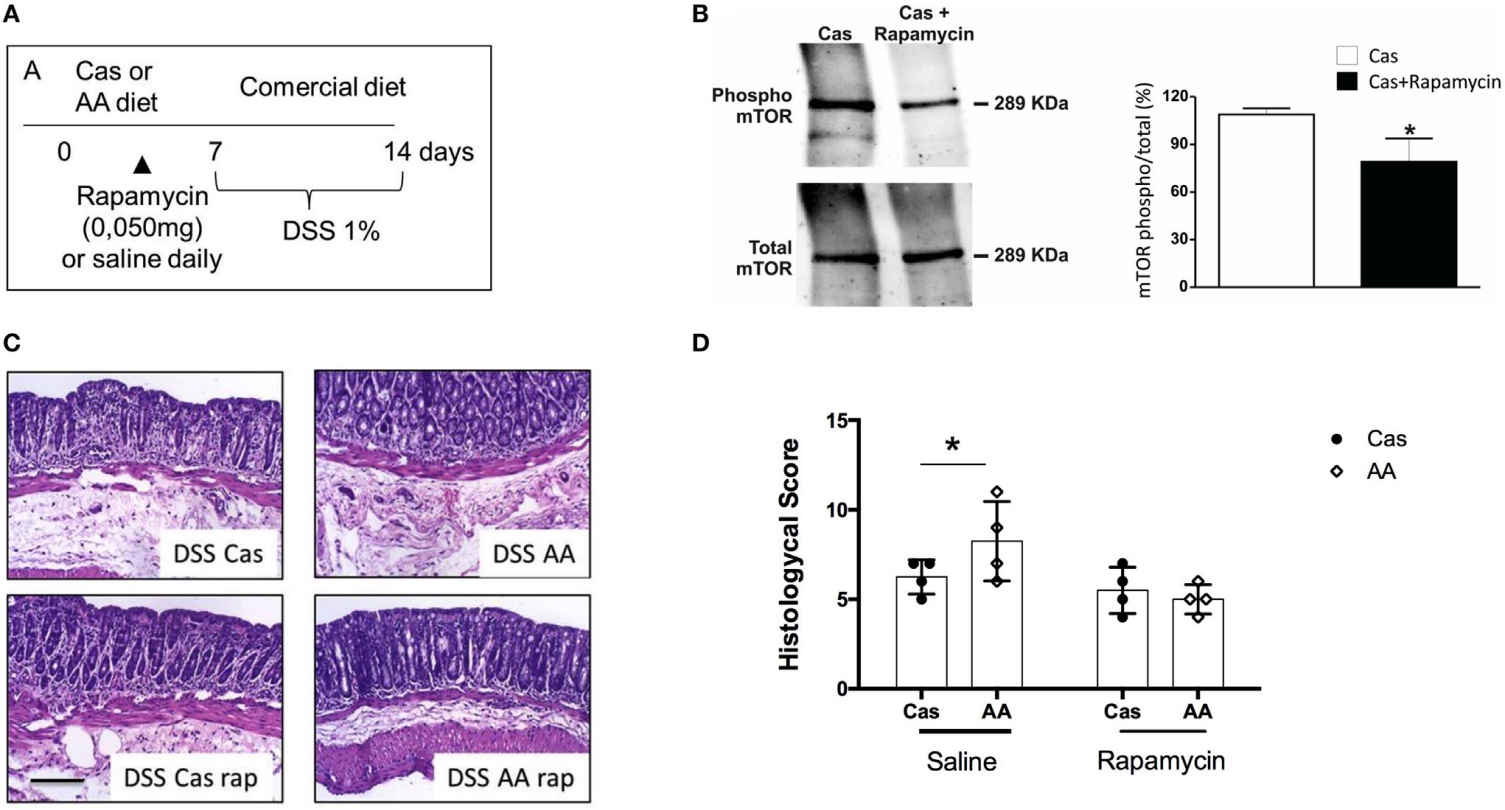
Effect of rapamycin administration during experimental diet consumption in colitis induction. **(A)** C57BL/6 mice at 7–8 weeks of age received either casein-containing diet (CAS) diet or diet containing free amino acids (AA diet) and a daily intraperitoneal (i.p.) dose of 10 mg/ml rapamycin for 7 days. Then, experimental diets were replaced by commercial mouse chow and colitis was induced by administration of 1% dextran sodium sulfate (DSS) for 7 days. During colitis development, mice received commercial chow. Expression levels of total and phosphorylated (pmTor) in colon samples of CAS-fed mice treated or not with rapamycin were analyzed by Western blotting **(B)**. Histological scores **(D)** were evaluated after colitis induction and representative images are shown **(C)**. Statistical analysis was performed between CAS-fed and AA-fed group at the end of each experimental time point using Student’s *t* test for parametric data and Mann–Whitney test for non-parametric data. *p* < 0.05.

To confirm that mTOR activation by amino acid containing diet (AA diet) was involved in exacerbation of colitis, we treated AA-diet-fed mice daily with the mTOR inhibitor rapamycin during diet consumption and previously to colitis induction by DSS (Figure 7A). First, we confirmed by western blot analysis, that mTOR phosphorylation (and activation) was blocked by rapamycin treatment (Figure 7B). Next, we observed that AA-diet-fed mice that received rapamycin during diet consumption showed no sign of colitis exacerbation suggesting that mTOR activation was involved in the mechanism by which AA diet triggered inflammation and exacerbation of colitis (Figures 7C–D).

## Discussion

The immunological role of dietary proteins has been already addressed by studies using either a standard rodent diet in which intact proteins were replaced by equivalent amounts of amino acids (7, 11) or by elemental diets (8, 28). Menezes et al. (7) showed that exposure to food proteins after weaning plays a physiological role in inducing the maturation of the immune system both locally and systemically (7). Furthermore, it was demonstrated that mice fed a diet containing free amino acids from weaning to adulthood are more susceptible to Leishmania infection (9) and more resistant to induction of nasal and oral tolerance (10, 11) as well as food allergy (11). In concert with these results, it has been shown recently that dietary proteins from solid food are responsible for the generation of the great majority of the small intestinal CD4+ Foxp3+ regulatory T cells involved in the induction of oral tolerance to food antigens (8). However, these studies have addressed the role of dietary proteins in the development of immunity from weaning to adulthood using an experimental diet containing free amino acids as a control that lacks antigenic proteins. Their general assumption was that addition of equivalent amounts of amino acids in replacement for proteins would only eliminate the antigenic impact of the intact molecules without yielding novel interferences in the immune activity. Therefore, the specific immunological effects of the free amino acids administered in the dietary composition have not been explored. Considering that elemental diets are used as complementary treatment for intestinal inflammatory diseases, clarifying these effects, especially in adult animals, will be important for the design of these nutritional therapeutic interventions.

In the present study, we showed that consumption of an AA diet resulted in immunological alterations in adult mice at steady state conditions. These alterations were not due to changes in their nutritional status. Consumption of AA diet was nutritionally equivalent to CAS diet concerning parameters such as weight maintenance and levels of total serum proteins, albumin, transferrin, glycemia, and triglycerides. Previous studies in humans and animals also showed nutritional equivalence between diets containing intact proteins and free amino acids (8, 11, 28, 29).

Consumption of AA diet resulted in several immunological changes including lower levels of serum IgG after 7 days of diet administration. Reduction in serum IgG and IgA levels have also observed when AA diets were consumed after weaning (7) and this effect was reversed by the introduction of a standard diet containing intact proteins during the period of 72 h (30). This suggests that exposure to dietary proteins stimulates proliferation and differentiation of B cells into Ig-producing plasma cells in lymphoid organs (30). In adult mice, removal of antigenic stimulation by dietary proteins was also able to promote reduction in IgG production, although this effect was less pronounced than in immature mice (7). Furthermore, the down modulation in IgG production caused by the absence of intact proteins was more important in the first week of dietary consumption for IgG. After 5 weeks, it seems that the presence of free amino acids in the diet had its own stimulatory effect on the B cells through other mechanisms. It is plausible that amino acids stimulate B cell proliferation by signaling directly through mTOR and, in later stages of AA diet consumption, this would compensate for the absence of antigenic proteins.

The dietary nitrogen source did not affect secretory IgA (SIgA) levels in the small intestine, but the consumption of diet containing free amino acids induced higher levels of this immunoglobulin in the colon after 5 weeks. It is likely that this is a direct result of the stimulatory effect of amino acids in immune cells as evidenced by the increase in cellular infiltration, reduction of goblet cells and presence of edema in the submucosal layer seen in histological examination of the colonic mucosa after 5 weeks of AA diet consumption. Moreover, AA-fed mice had an augmented frequency of proliferating cells in the small intestine 1 week after diet consumption. Higher sIgA levels were also observed in AA-fed mice during intestinal inflammation. Other studies have also shown that gut inflammation in mice (31) and humans (32) is associated with augmented production of secretory IgA. Of course, we cannot rule out the possibility that changes in the microbiota of AA-fed mice also contributed to all these alterations including the increase of SIgA in the colon at steady state and during colitis. Several studies have shown that the composition of the diet can affect both the diversity and the number of intestinal bacteria (33). Dietary therapy with either SCD (16) or exclusive enteral nutrition (EEN) with elemental formula are reported to improve dysbiosis associated with Crohn’s disease and ameliorate intestinal inflammation in affected patients (17). Elemental diet consumption has also been shown to impact in microbiota composition in mice (34). The intestinal microbiota, in turn, can modulate the intestinal levels of SIgA (35). Appendectomized mice, for instance, which have low levels of SIgA in the colon, presented altered composition of fecal microbiota (36).

Consumption of AA diet resulted in an increase in the number of lamina propria cells of the small intestine, but not of the colon. Since amino acids are known to signal through intracellular receptors such as mTOR that control cell proliferation, we examined whether AA-fed mice had increased number of proliferating cells in the small intestinal mucosa using an anti-PCNA monoclonal antibody. AA-fed mice had an augmented number of PCNA+ cells in the small intestine than control CAS-fed mice suggesting that ingestion of free amino acids had an effect in cell proliferation in the gut mucosa. T cell subsets in the small intestine were specifically affected by AA diet. Numbers of total and activated (CD44+) CD4+ T cells as well as Th17 (CD4+ RORγt+) cells were augmented in the small intestine, but not in the colon, of AA-fed mice. It is known that naïve CD4+ T lymphocytes can differentiate into Th17, Th1, Th2, or Treg cells depending on the cytokines present during their activation. Differentiation into Th17 cells requires the expression of the transcription factor RORγt which is upregulated in the presence of TGF-β and IL-6 (37). We observed an increase in IL-6 and TGF-β levels in intestinal extracts isolated from AA-fed mice after 1 week of diet consumption which may have contributed to the increase in the number of CD4 + RORγt + T cells observed 4 weeks later in the small intestine. However, AA-fed mice had higher IL-17A concentrations in the intestine as early as 1 week of diet consumption. Other cell types can produce IL-17, including γδ T cells, ILC3, natural killer cells, macrophages, and Paneth cells (38, 39). The role of dietary protein and amino acids in the proliferation and differentiation of these cell types need to be evaluated. Of note, colonic levels of IL-10 were also increased in AA-fed mice. Since IL-10 is an important cytokine for gut homeostasis (40), its upregulation may help to control local inflammatory events and to prevent the outburst of a spontaneous colitis. Indeed, intestinal permeability was higher in AA-fed mice after 5 weeks of diet consumption and histological analysis of the large intestine showed signs of inflammatory events, but no overt inflammation was detected.

It is remarkable that AA diet consumption by adult mice led to histopathological changes mostly in their colonic mucosa suggesting that prolonged consumption of diets containing free amino acids can be especially harmful for the final portions of the intestine. Although the small intestine was also affected in these mice, our hypothesis is that intrinsic features of the colonic mucosa makes it more susceptible to inflammatory changes brought about by amino acid ingestion. Since the concentration of autochthonous bacteria in the colon exceeds by large the one in the small intestine, this portion is physiologically primed and more prone to undergo pathological alterations once stimulated by a proinflammatory stimuli.

To further explore the effects of amino acid ingestion in the colon, we examined the impact of AA diet during DSS-induced colitis. Mice were fed AA diet concomitantly to the induction of colitis. AA diet consumption significantly boosted colitis development when three cycles of DSS were administered. AA-fed mice had higher clinical scores and weight loss throughout disease development including at the remission time points that followed each cycle of DSS. The histological index determined after the third cycle was also higher in AA-fed mice when compared to CAS-fed mice. Histological evaluation showed a more intense inflammatory infiltrate in the mucosa and submucosa, loss of mucosal architecture and thickening of the muscle layer. Furthermore, AA diet consumption prior to colitis induction also led to a more severe inflammation of the colonic mucosa. This suggests that the effects induced by dietary free amino acids at steady state exacerbated the development of an experimental IBD and that they would last even after discontinuation of diet consumption.

Elemental diets, which have in its composition only free amino acids, are used in the nutritional treatment of patients with IBDs in case of polymeric diet intolerance. Some studies have shown that elemental diets yielded more beneficial results in active Crohn’s disease (CD) (17) even when compared to polymeric diets (41) and treatment with corticosteroids (42, 43). Others showed no difference between the formulas used in the treatment of active CD (44, 45). Finally, some authors have demonstrated that polymeric formulations are the most suitable option since they have the same effects on remission rate as elemental diets, but promote greater weight gain (46). Therefore, polymeric and oligomeric diets are preferred over elemental diets although the mechanisms involved in the differential effects of amino acid versus intact proteins during IBD are still elusive (12–14).

There are few studies evaluating the effects of elemental diets in experimental models of colitis. In the colitis model induced by transferring cells from IL-10-deficient C57BL/6 mice to CB-17 SCID mice, elemental diet consumption prevented weight loss and suppressed intestinal inflammation, whereas mice fed standard diet showed a severe form of colitis (34). However, elemental and polymeric diets used in this study had alterations in other nutrients in addition to the replacement of nitrogen source by amino acids. Therefore, the effects observed were a net result of specific changes in the whole dietary composition. In this study, we examined the exclusive and specific role of stimulation by amino acids versus dietary proteins in colitis development.

One major factor to be considered when using diets containing a high concentration of free amino acids would be the impact of its increased osmolarity on intestinal absorption and permeability. Increased gut permeability is known to play a key role in triggering bacterial translocation and inflammation during IBDs (47). Indeed, we observed that AA-fed mice had an augmented intestinal permeability when tested for the absorption of radiolabeled DTPA and this defect in the gut barrier function could be an important trigger of colonic inflammation.

Another mechanism by which AA fed diet could contribute to gut inflammation would be a direct effect of amino acids in the immune cells located in the gut mucosa. The increase in the number of total cells, CD4+ T lymphocytes, CD4+ CD44+ activated T cells and Th17 cells observed in the *lamina propria* of the small intestine of AA-fed mice at steady state suggested that the AA diet had an effect either in T cell activation and proliferation or in the recruitment of T cells to the intestine. This effect was not observed in the colon suggesting that it was restricted to the portion where digestion and absorption of proteins take place. Free amino acids are signaling molecules and can modulate the activity of immune cells through pathways such as the one driven by the mTOR. mTOR is a conserved serine/threonine kinase that has a central role in the regulation of cell growth and metabolism, sensing, and integrating diverse environmental signals, including nutrients and growth factors (48, 49). mTOR signals through two distinct multiprotein complexes called mTOR complex (mTORC)1 and mTORC2 which are sensitive and insensitive to rapamycin, respectively. The mTORC1 senses the changes in the nutritional environment, including changes in amino acid concentration (50) mainly for l-glutamine and l-asparagine and other essentials amino acids such as l-leucine, l-tryptophan, l-phenylalanine, and l-arginine (51). Signaling through mTORC1 contributes to maintain homeostasis of the intestinal epithelium (52, 53) and regulates various cells of the innate and adaptive immune system (54) such as dendritic cells (55), macrophages (52), neutrophils (56), and regulatory T cells (57). The specific role of mTOR activation by diets containing free amino acids in the modulation of immune cells during dietary consumption is still unclear.

To address the putative role mTOR activation in the inflammatory effects triggered by the AA diet, we used rapamycin during AA diet consumption prior to colitis induction. Several studies have demonstrated that blockade of mTOR activation by inhibitors such as rapamycin and everolimus reduces experimentally induced colitis. Rapamycin forms a gain-of-function complex with the intracellular FK506-binding protein protein, and this complex directly interacts with and inhibits mTOR (58). Administration of everolimus to IL-10-deficent mice, which develops spontaneous colitis, inhibits the proliferation of activated T cells, decreases the number of Th1 cells and the production of inflammatory cytokines by Th1 cells in the *lamina propria*, and ultimately ameliorates chronic colitis (59). In a model of DSS-induced colitis, rapamycin administration decreased leukocyte migration and effectively reduced colonic inflammation (59, 60). These data suggest that increase in mTOR activation by dietary amino acids may be involved in the increased colitis severity observed in AA-fed mice. Indeed, our results showed that blocking mTOR phosphorylation (and activation) by rapamycin treatment concomitantly to AA diet consumption inhibited its boosting effect in colitis development.

In conclusion, our study demonstrates that the consumption of a diet containing free amino acids caused several immune alterations in the intestinal mucosa including increase in SIgA and cytokine secretion in the colon, augmented intestinal permeability and accumulation of proliferating cells and activated CD4+ T lymphocytes in the small intestine. Although these alterations did not lead to spontaneous development of colitis, free amino acids in the diet caused exacerbation of intestinal inflammation when consumed either during or prior colitis development. Blockage of mTOR activation by treatment with rapamycin during AA diet consumption prevented colitis exacerbation suggesting this cellular sensor complex was involved in the effect mediate by the AA diet. These results provide further insight into the immunological effects of elemental versus polymeric diet formulations used as a complementary therapeutic tool for IBDs.

## Author Contributions

AS and SFA performed the experiments, analyzed the data, and wrote the article; MGM, LL, and MFG performed the experiments and helped with the analysis of data; DR helped with the experiments on flow cytometry; PVB performed the gut permeability analysis; EV and EF performed the cell proliferation analysis; TC and FR performed the blots for mTOR analysis; DC performed the histological analysis and helped discussing the data; AG-S helped designing the experiments and analyzing the data; ACF designed the experiments, supervised the study, analyzed the data, and wrote the manuscript.

## Conflict of Interest Statement

The authors declare that the research was conducted in the absence of any commercial or financial relationships that could be construed as a potential conflict of interest.

## Supplementary Material

Figure S1 Body weight, food consumption and biochemical tests. C57BL/6 mice at 720138 weeks of age were fed either experimental diet (AA diet) or control diet (CAS diet) for 5 weeks. Body weight **(A)** and food consumption **(B)** were evaluated during the experimental protocol. Biochemical tests for total serum proteins **(C)**, albumin **(D)**, glycemia **(E)**, triglycerides **(F)**, and transferrin **(G)** were performed at the end of first, third, and fifth week, *n* = 5–10. Statistical analysis was performed between CAS-fed and AA-fed group at the end of each experimental time point using Mann Whitney test. *p* < 0.05.

Figure S2 Cytokine levels in spleen. C57BL/6 mice at 7–8 weeks of age were fed either experimental diet (AA diet) or control diet (CAS diet) for 5 weeks. Levels of IL-10, IL-4, IFN-γ, IL-17A, TGF-β, IL-6, and IL-12 were measured in the spleen by ELISA after 7 days of dietary consumption, *n* = 4–7. Statistical analysis was performed between CAS-fed and AA-fed group at the end of each experimental time point using Student’s *t* test for parametric data. *p* < 0.05.
