# Supplementary figures and images for: Consumption of Diet Containing Free Amino Acids Exacerbates Colitis in Mice
Source: Front Immunol. 2017 Nov 20;8:1587. doi: 10.3389/fimmu.2017.01587 (PMC5701921; doi:10.3389/fimmu.2017.01587)

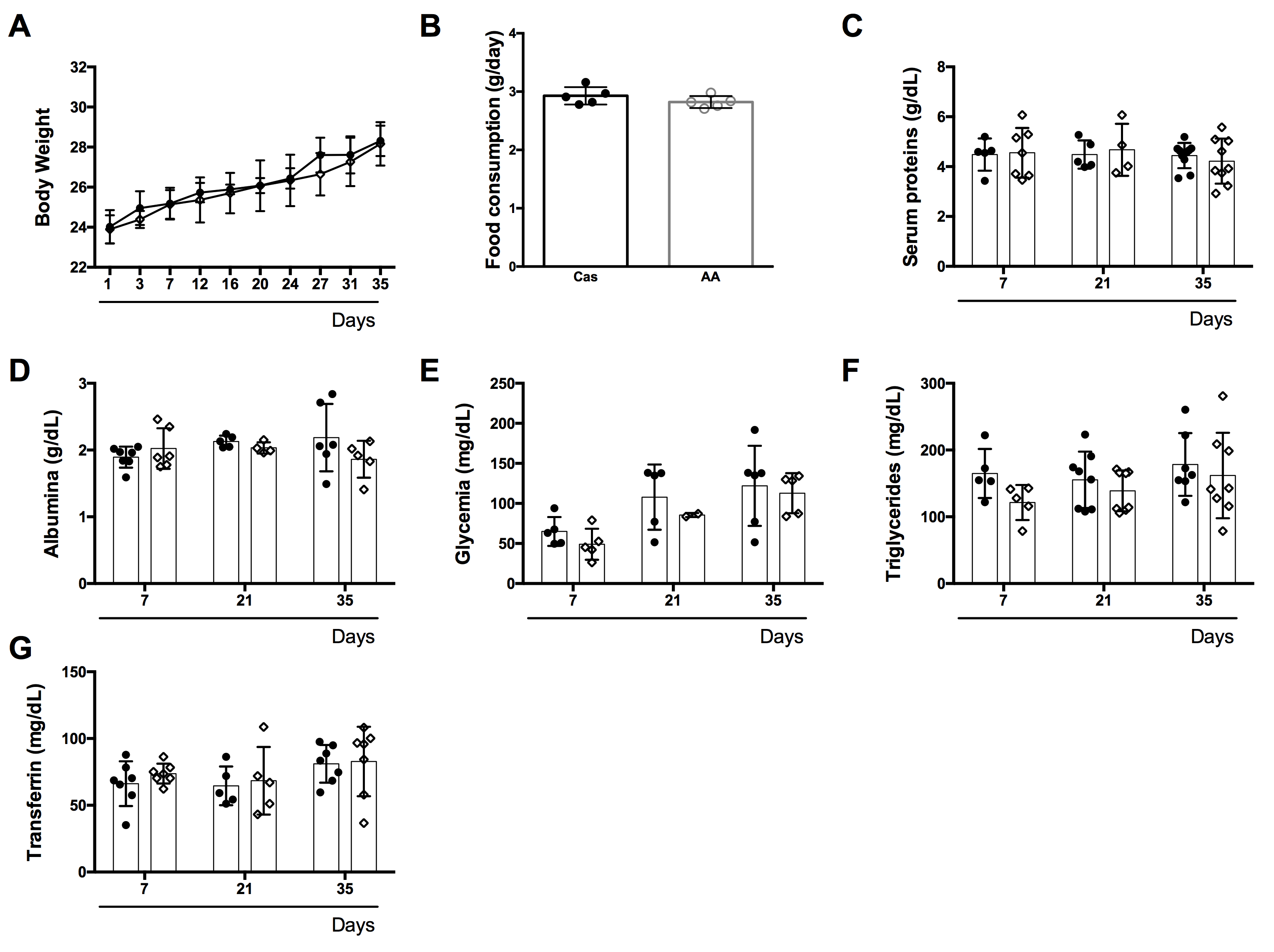

Supplement: Figure S1 — Body weight, food consumption and biochemical tests. C57BL/6 mice at 720138 weeks of age were fed either experimental diet (AA diet) or control diet (CAS diet) for 5 weeks. Body weight (A) and food consumption (B) were evaluated during the experimental protocol. Biochemical tests for total serum proteins (C), albumin (D), glycemia (E), triglycerides (F), and transferrin (G) were performed at the end of first, third, and fifth week, n = 5–10. Statistical analysis was performed between CAS-fed and AA-fed group at the end of each experimental time point using Mann Whitney test. p < 0.05. [file image_1.tiff]

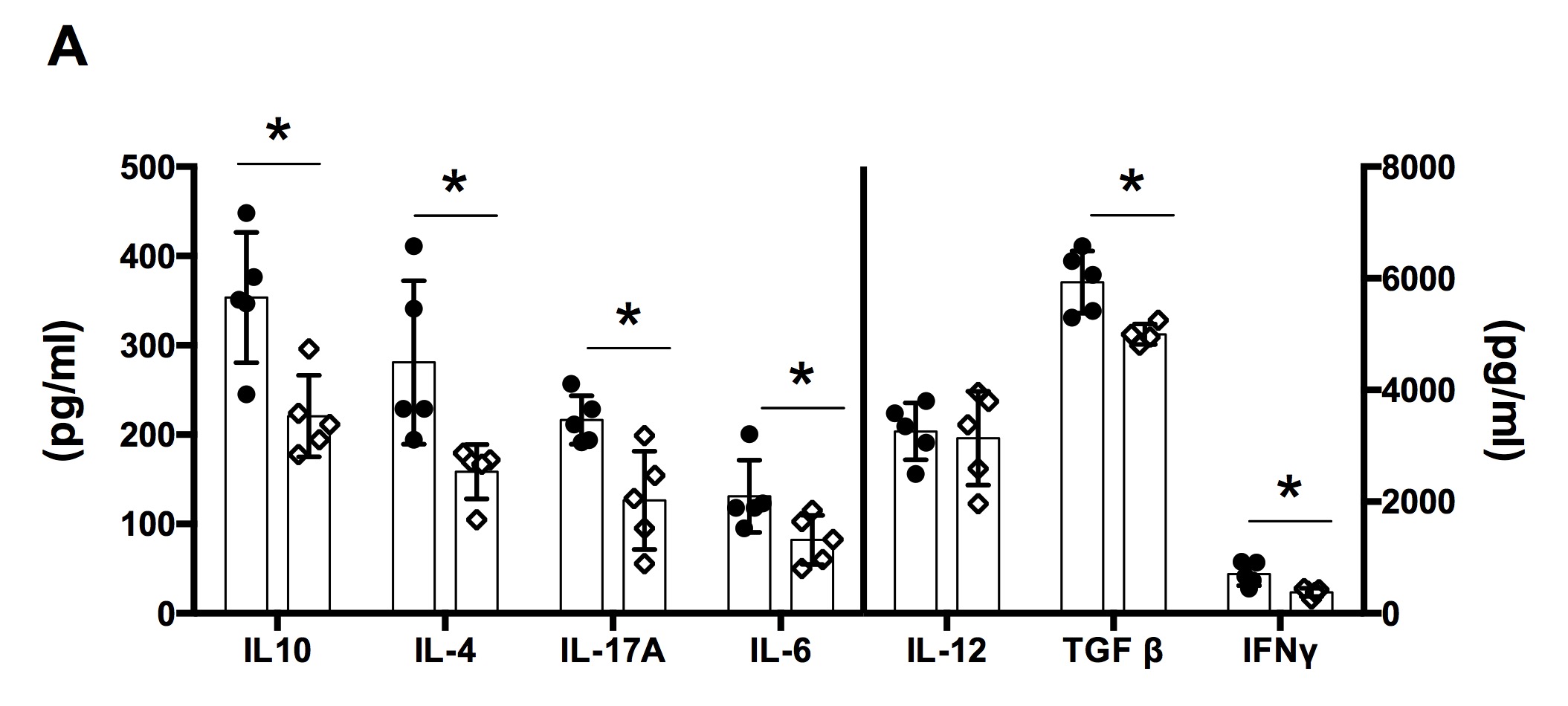

Supplement: Figure S2 — Cytokine levels in spleen. C57BL/6 mice at 7–8 weeks of age were fed either experimental diet (AA diet) or control diet (CAS diet) for 5 weeks. Levels of IL-10, IL-4, IFN-γ, IL-17A, TGF-β, IL-6, and IL-12 were measured in the spleen by ELISA after 7 days of dietary consumption, n = 4–7. Statistical analysis was performed between CAS-fed and AA-fed group at the end of each experimental time point using Student’s t test for parametric data. p < 0.05. [file image_2.jpeg]
